# Reproductive asynchrony within social groups of female eastern wild turkeys

**DOI:** 10.1002/ece3.10171

**Published:** 2023-06-14

**Authors:** Erin E. Ulrey, Michael J. Chamberlain, Bret A. Collier

**Affiliations:** ^1^ School of Renewable Natural Resources Louisiana State University Agricultural Center Baton Rouge Louisiana USA; ^2^ Warnell School of Forestry and Natural Resources University of Georgia Athens Georgia USA

**Keywords:** asynchronous, breeding, dominance, *Meleagris gallopavo*, nesting, social hierarchies, wild turkey

## Abstract

Coordination in timing of reproduction is driven by multiple ecological and sociobiological processes for a wide array of species. Eastern wild turkeys (*Meleagris gallopavo silvestris*) use a male dominance polygynous mating system, where males communicate with females via elaborate courtship displays and vocalizations at display sites. Most females prefer to mate with dominant males; therefore, asynchronous breeding and nesting may occur which can disproportionately influence individual fitness within breeding groups. For female wild turkeys, there are reproductive advantages associated with earlier nesting. As such, we evaluated reproductive asynchrony within and between groups of GPS‐tagged female eastern wild turkeys based on timing of nest initiation. We examined 30 social groups with an average of seven females per group (range 2–15) during 2014–2019 in west central Louisiana. We found that the estimated number of days between first nest initiation across females within groups varied between 3 and 7 days across years, although we expected 1–2 days to occur between successive nesting attempts of females within groups based on observations of captive wild turkeys in the extant literature. The number of days between successive nest attempts across females within groups was lower for successful than failed attempts, and nests with an average of 2.8 days between initiation of another nest were more likely to hatch. Our findings suggest that asynchronous reproduction may influence reproductive success in female wild turkeys.

## Graphical abstract

Eastern wild turkeys (*Meleagris gallopavo silvestris*) exhibit social hierarchies wherein dominance is established through agnostic interactions within social groups. We evaluated reproductive synchrony within and between presumed social groups of GPS‐tagged female eastern wild turkeys by inferring female social rank based on timing of nest initiation. Our findings suggest that social hierarchies may influence reproductive success in female wild turkeys, and we postulate that social constraints could cause variation in timing of nest initiation for females within social groups.

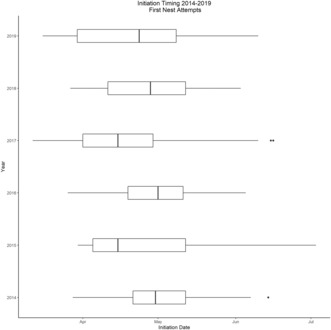

## 1 INTRODUCTION

Multiple ecological and sociobiological processes drive the coordination of timing of reproduction for a wide array of species (Lack, 1968; Perrins, 1970). Matching reproductive activities with optimal environmental conditions is crucial to optimize reproductive success (Ims, 1990a). As such, temporal clustering of reproductively active individuals is typically driven by climatic seasonality (Ims, 1990a), especially when breeding seasons are restricted to shorter temporal periods (Emlen & Demong, 1975; Findlay & Cooke, 1982). In local regions, the availability of food, temperature, and rainfall may modify the timing of reproduction (Wingfield & Kenagy, 1991). Photoperiod is critical to the timing of seasonal processes (Gwinner, 2003) and regulates endogenous rhythms within individuals which can be modified by social cues, such as estrus synchrony in mammals among group living females (Ims, 1990a). Through signals exchanged between individuals within a population, the availability of social information can influence temporal clustering (Helm et al., 2006) of reproductive events (Gochfeld, 1980; Lack, 1968).

Temporal patterns of breeding events within a population may influence the type of mating system. Monogamous species regularly demonstrate a high degree of temporal clustering (Emlen & Oring, 1977; Gochfeld, 1980), and in monogamous systems, male investment in courtship activities will limit opportunity for extra‐pair reproductive activities (Grant & Kramer, 1992; Westneat, 1992) with the consequence being synchronized reproductive activities (Knowlton, 1979). Colonial birds demonstrate high degrees of clustered nesting (Darling, 1938; Gochfeld, 1980; Lack, 1968), as individuals synchronize reproduction to simultaneously reproduce (Gochfeld, 1980) by initiating laying over a period of several days (Patterson, 1965), resulting in higher rates of nest success (Di Maggio et al., 2013) by reducing offspring mortality (Darling, 1938). However, when predatory species have a generalist functional response (Smouth et al., 2010), populations exhibiting synchronized reproduction will experience increased predation, and asynchronous reproduction may then be the best reproductive strategy for a population (Ims, 1990b).

An important determinant of a mating system is the spatial distribution of sexually receptive females (Ims, 1987). A polygynous mating system will likely result when females are spatially clumped with territorial males defending these groups, as males are able to effectively monopolize access to females (Baird & Leibold, 2023; Post, 1992; Shuster & Wade, 2003; Webster, 1994). When female reproduction is asynchronous in polygynous systems, females can choose between more males (Wells, 1977), typically creating a male reproductive skew (Emlen, 1976; Mackenzie et al., 1995), disproportionately affecting the fitness of individuals and increasing the opportunity for optimal mate choice.

The eastern wild turkey (*Meleagris gallopavo silvestris*; hereafter, wild turkey) uses a male dominance polygynous mating system where males communicate with females via elaborate courtship displays and vocalizations at display sites (Chamberlain et al., 2018; Krakauer, 2005; Wakefield et al., 2020). Female wild turkeys visit display areas for the purpose of mating, while assessing males and selecting preferred mates. Male wild turkeys do not provide resources to female wild turkeys other than gametes and do not provide parental care to young (Buchholz, 1997; Krakauer, 2008; Robel, 1970).

Within polygynous mating systems, a limited number of males are required to guarantee fertilization (Haigh & Hudson, 1993), and a single male is capable of inseminating a large number of females within a short period of time (Jabbour et al., 1991; Skjenneberg & Slagsvold, 1979). Dominant male wild turkeys breed whereas non‐dominant and juvenile males seldom do (Healy, 1992), and the presence and behavior of dominant males can suppress physiological and behavioral development of subdominants (Lisano & Kennamer, 1977). Therefore, to optimize reproductive success females are more likely to mate with dominant males within breeding groups (Healy, 1992; Krakauer, 2008; Watts & Stokes, 1971).

Wild turkey flocks maintain home ranges but are not territorial. Male and female wild turkeys maintain separate dominance hierarchies, and these hierarchies remain stable unless dominant birds suffer mortality (Healy, 1992; Watts & Stokes, 1971). Female wild turkeys feed and congregate with other females throughout the laying period; however, these social activities occur away from their nest sites (Conley et al., 2016; Williams Jr. et al., 1974). Within groups, it may benefit individual females to nest earlier than conspecifics, because female wild turkeys that nest earlier in the reproductive season may experience fitness advantages associated with preferential nest site selection and increased length of development for young (Robel, 1970; Robel & Ballard Jr., 1974). As such, we evaluated reproductive synchrony within and across groups of GPS‐tagged female Eastern wild turkeys (Cristol, 1995; Healy, 1992; Schmutz & Braun, 1989; Watts & Stokes, 1971). We hypothesized that the male dominance polygynous mating system used by wild turkeys would influence the timing of reproduction and influence individual reproductive success. Specifically, we predicted that within groups, females would initiate laying of their nests asynchronously. We also predicted that females who initiated nests earlier in the reproductive season and failed would be more likely to initiate subsequent renest attempts before conspecifics making their first nest attempt. Lastly, we predicted that earlier nesting females would travel shorter distances within their reproductive ranges prior to onset of nest initiation compared to later nesting females.

### 1.1 Study area

We conducted research on the Kisatchie National Forest (KNF) and Peason Ridge Wildlife Management Area (WMA) in west central Louisiana. The KNF was owned and managed by the United States Forest Service (USFS) and divided into five Ranger Districts. We conducted research on the Catahoula Ranger District, Kisatchie Ranger District, Winn Ranger District, and the Vernon unit of the Calcasieu Ranger District located in Grant, Natchitoches, Winn, and Vernon parishes, respectively. Peason Ridge WMA was jointly owned by the USFS and the US Army. The spatial area of Catahoula Ranger District, Kisatchie Ranger District, Winn Ranger District, Vernon unit, and Peason Ridge was approximately 49,169, 41,453, 67,408, 61,202, and 74,309 ha, respectively. Our study sites were composed of pine‐dominated forests encompassing rolling hills, high ridges, and sandy creek bottoms. Vegetative communities consisted of loblolly pine (*Pinus taeda*), longleaf pine (*P. palustris*), shortleaf pine (*P. echinata*), slash pine (*P. elliotti*), mixed pine‐hardwood forests, and hardwood riparian areas. Our sites contained forest openings, utility rights‐of‐way, and forest roads distributed throughout. Rural infrastructure, agricultural fields, pasture, and privately owned industrial forest lands bordered our study sites. Prescribed fire was applied on a 3–5‐year return interval. The study sites experienced subtropical climates with mean daily temperatures ranging from a low of 9.4°C in January to a high of 28.3°C in July and mean rainfall averaged ~114 cm.

## 2 METHODS

### 2.1 Capture and handling

We captured female wild turkeys opportunistically in flocks ranging from three to >20 using rocket nets baited with corn from January to March 2014–2019. We aged each individual using the presence of barring on the 9th and 10th primaries (Pelham & Dickson, 1992). We fitted all females with a uniquely identifiable aluminum rivet tarsal band and GPS/VHF transmitter (89 g; Biotrack Limited; Guthrie et al., 2011). Transmitters were attached backpack style using marine grade shock cord (3 mm), which typically degraded after several years and fell off the birds if they were not recovered dead. We programmed GPS units to collect data at 1‐h intervals (Cohen et al., 2018) between 05:00 and 20:00 daily with one location at night (23:59:58) to identify roosts until the battery died or the unit was recovered. We immediately released individuals at the capture location following processing. Capture, handling, and marking procedures were approved by the Louisiana State University Agricultural Center Animal Care and Use Committee (Permits A2015‐07 and A2018‐13). We monitored live–dead status daily during the reproductive season using handheld Yagi antennas and Biotracker receivers (Biotrack Ltd.). We downloaded GPS locations once per week via a VHF/UHF handheld command unit receiver (Biotrack Ltd.).

### 2.2 Group definition

Wild turkeys flock together in groups of adult males, juvenile males, or females. We assumed that females within a specific area had access to the same resources and presumably the same preferred males. Based on estimates of daily movements by females (Bakner et al., 2019; Conley et al., 2016), we considered individuals captured within 2 km of each other to use the same area as these individuals appeared to regularly interact and at further distances the probability of overlap with other flocks was low (0.02; Niedzielski & Bowman, 2016, Schofield, 2019). To further ensure we accurately defined groups, we used a dynamic Brownian Bridge movement model (dBBMM) to create 99% utilization distributions (UDs) for each individual (Byrne et al., 2014) for the 21 days before the first female in each group laid the first egg at an eventual nest site. We chose a 21‐day period because we were interested in overlap in space use during the time immediately preceding initiation of the first nest in the group (Watts & Stokes, 1971). We calculated all UDs in program R version 3.2.5 (R Core Team, 2020) using package move (Kranstauber & Smolla, 2013). We used a window and margin size equal to 21 and 9, respectively, and a location error of 10 m (Byrne et al., 2014). Individuals that share space may constitute a single social unit (Brown, 1975), therefore we calculated the percentage of female UDs that intersected at least one other female's unique UD within a defined group during the 21‐day period to quantify shared space use (Kernohan et al., 2001). If a female's UD intersected any another female's UD within their group, we considered those females to share space and to likely have access to the same resources. We also assumed that females with overlapping UDs are likely to mate with the same dominant males, as females often move in loose groups on display sites (Höglund et al., 1990; Watts & Stokes, 1971).

### 2.3 Nest monitoring

We determined locations of each nesting attempt for each female when an individual's locations became concentrated around a single point for several days (Conley et al., 2015; Guthrie et al., 2011; Wood et al., 2019; Yeldell et al., 2017). We defined the first date of nest incubation as the first day we recorded the nightly roost location at the nest site, indicating the female continued incubation during the night (Bakner et al., 2019). To determine the first date of egg laying (hereafter nest initiation), we evaluated GPS locations to determine when a female initially visited the nest site as female wild turkeys do not visit their nest site until they lay their first egg (Collier et al., 2019; Conley et al., 2016). We monitored each nesting attempt following Bakner et al. (2019) and after nest termination, located nest sites using VHF telemetry and GPS data to confirm the nest location and determine nest fate. We considered a nest to have been depredated or abandoned if the female left the nest ≤25 days into incubation, or if only intact eggs, no eggs, or egg fragments were found at the nest bowl. We considered a nest successful if ≥1 live poult hatched and was confirmed visually during subsequent brood surveys following methods outlined in Chamberlain et al. (2020).

We scaled the initiation date of the first nest attempt to group, where the date of the first nest initiation was noted as Day 1. We delineated subsequent nest attempts based on the number of days after the first nest was initiated. We subtracted the initiation day of the second nest from the initiation day of the first nest and then subtracted the initiation day of the third nest from the initiation day of the second nest, and so on for each first nest attempt within each group. We then calculated mean number of days between each nest initiation attempt within each group. We speculated that groups with more individuals would have more days between subsequent nest attempts compared to smaller groups. Presumably, larger groups would contain more females competing to copulate with dominant males (Orbach et al., 2015), whereas smaller groups would have less competition and thus be able to copulate in a shorter temporal window, resulting in a narrower time window during which nests were initiated by females in that group (Avery, 1984; Dewsbury, 1982; Foster, 1983; Gratson et al., 1991; Möller, 1992; Trail, 1985).

### 2.4 Analysis of reproductive timing

Female wild turkeys that attempt reproduction earlier within a season are expected to have greater annual reproductive success compared to later breeding individuals (Crawford et al., 2021; Keever et al., 2022). Females can presumably select nest sites that could confer fitness advantages through improved nest success (Crawford et al., 2021; Keever et al., 2022; Martin, 1995a, 1995b; Sӕther, 1990), compared to females that nest later and may be forced to nest in suboptimal parts of their ranges or travel farther distances to find suitable nest sites (Badyaev et al., 1996; Schaap et al., 2005). To test the prediction that females that initiated nests earlier would travel shorter distances within their ranges prior to onset of nest initiation, we used the distance between a female's nest location and the centroid of the UD range of the 21‐day period before the first nest of each group was initiated as our metric. We measured the distance between the centroid of each female's 99% UD range to each of her nest attempts in ArcGIS 10.6 (Environmental Systems Research Institute, Inc.). To locate the centroids of each 99% UD, we calculated the *x* and *y* centroids of each UD in the attribute table. We then created a line between each nest attempt and the centroid and calculated the distance between each nest attempt and the centroid within the 99% UD. To test for differences in mean distance traveled for females with successful versus failed nests, we used an independent two‐group *t*‐test with an α = .05 in R (R Core Team, 2020). Likewise, we used a binomial generalized linear model (GLM) in R (R Core Team, 2020) to estimate nest success as a function of first nest initiation date. We then used a Poisson GLM to estimate the rate (in days) at which females initiated their first and second nesting attempts as a function of group size and year. Finally, we used linear regression to evaluate the effect of group size on the number of days between nesting attempts within groups.

## 3 RESULTS

**FIGURE 1:**
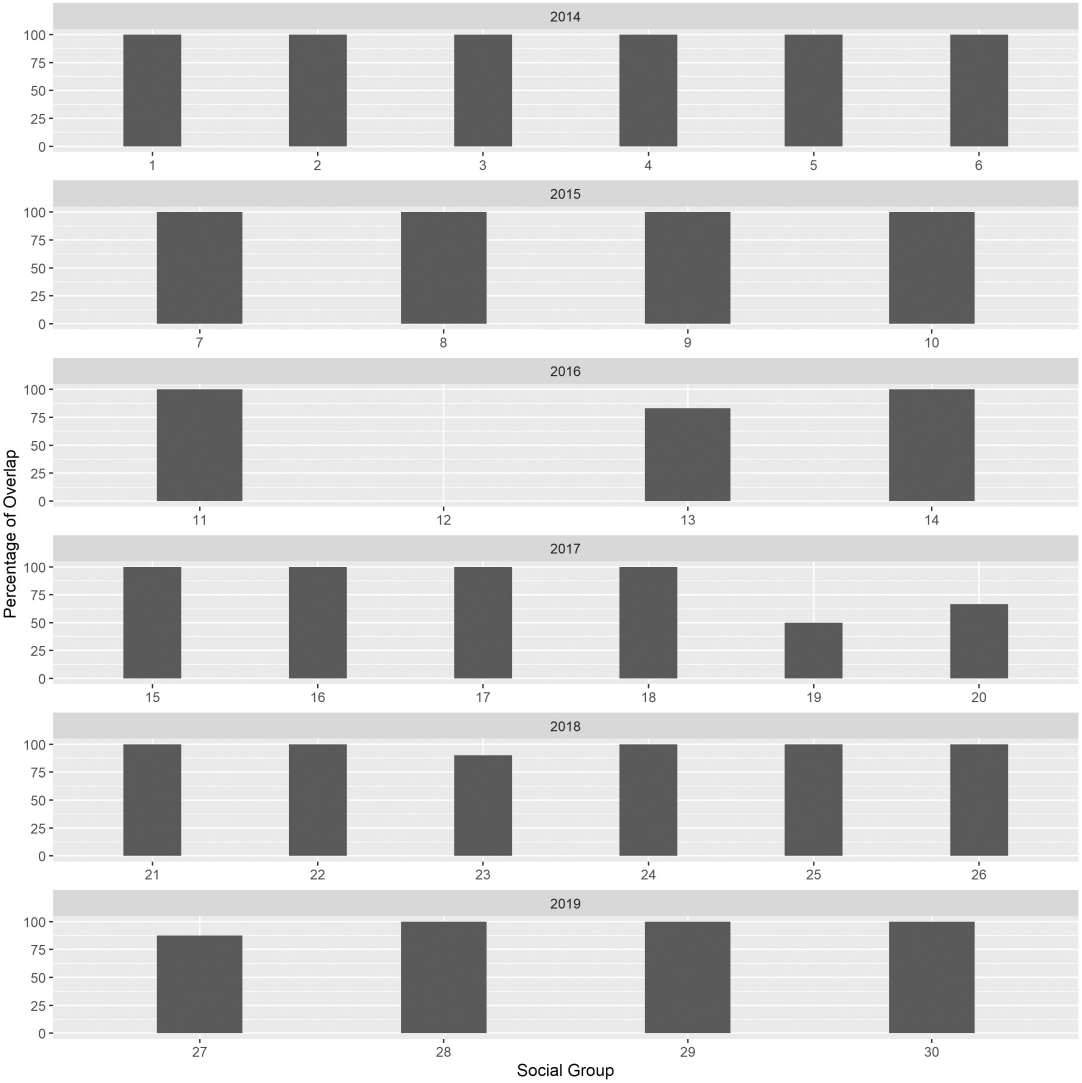
Proportion of female wild turkey ranges that overlapped ≥1 other female's range within their respective social groups during the 21‐day period prior to initiation of first nest attempts on Kisatchie National Forest and Peason Ridge Wildlife Management Area, west central Louisiana, USA during 2014–2019.

We captured and radio‐marked 225 female turkeys (201 adults, 24 juveniles) during 2014–2019. We monitored 245 nesting attempts (158 first, 69 second, 17 third, and 1 fourth nest attempt, respectively) from 158 females and identified 30 groups with an average of seven females per group (Table 1). Across all groups and years, mean proportion of individual ranges during the 21‐day period prior to first nest initiation that did not overlap was 7.18% (Figure 1). Within groups, we observed that ≥80% of females‐maintained ranges that overlapped >1% during the 21‐day period prior to nest initiation (Figure 1). Mean distance from the 21‐day range centroid to the subsequent nest location ranged from 974 to 6403 m and averaged 2107 m (SD = 2131) across all females. Mean distance from the centroid within the 21‐day range to the first nest attempt for successful (mean = 1743 m, SD = 1175) and unsuccessful nests (mean = 2154 m, SD = 2236) was similar (*t* = −1.28, df = 46, *p* = .205).

**TABLE 1:** Number of female eastern wild turkeys (*Meleagris gallopavo silvestris*) within each group (No.), mean day of first nest initiation, and range of first nest initiation days (Range) with associated standard deviations (SD) where Day 1 is the first nest initiation by a female within a social group on Kisatchie National Forest and Peason Ridge WMA in west central Louisiana during 2014–2019.

Year	Group	No.	Mean day (SD)	Range
2014	1	9	7.2 (5.1)	1–13
	2	7	17 (17.5)	1–39
	3	7	7.75 (5.9)	1–15
	4	3	5.5 (6.4)	1–10
	5	2	5 (5.7)	1–9
	6	4	4.33 (3.5)	1–8
2015	7	7	21.25 (17.2)	1–40
	8	4	5.67 (6.4)	1–13
	9	3	7 (8.5)	1–13
	10	8	17 (8.7)	1–27
2016	11	9	23.4 (16.2)	1–43
	12	3	15.5 (20.5)	1–30
	13	9	16 (10.4)	1–23
	14	6	17.67 (14.5)	1–27
2017	15	13	14.82 (7.9)	1–28
	16	12	16.1 (15.1)	1–47
	17	5	14.6 (9.1)	1–25
	18	9	14.83 (8.5)	1–26
	19	3	12.33 (10.3)	1–21
	20	9	10.17 (8.5)	1–22
2018	21	6	13.2 (10.1)	1–29
	22	13	24.43 (16.0)	1–43
	23	11	15.88 (13.4)	1–38
	24	3	11.5 (14.9)	1–22
	25	15	17.17 (14.0)	1–43
	26	3	13.5 (17.7)	1–26
2019	27	10	17.28 (11.4)	1–35
	28	7	18.57 (23.2)	1–66
	29	7	15.83 (14.1)	1–40
	30	9	14 (15.3)	1–39

**FIGURE 2:**
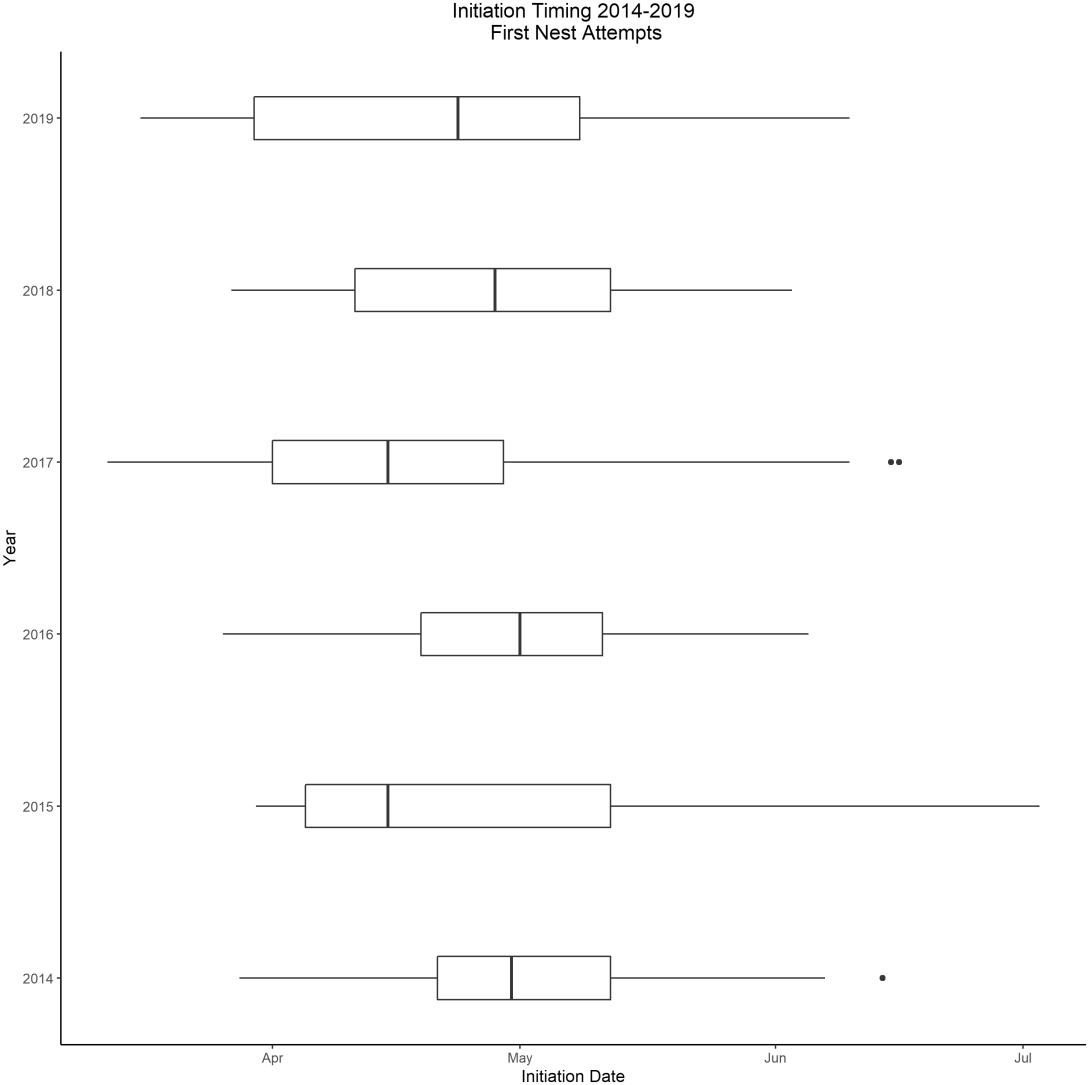
Boxplot of first nest initiation dates of female eastern wild turkeys (*Meleagris gallopavo silvestris*) during 2014–2019 on Kisatchie National Forest and Peason Ridge Wildlife Management Area, west central Louisiana, USA.

**FIGURE 3:**
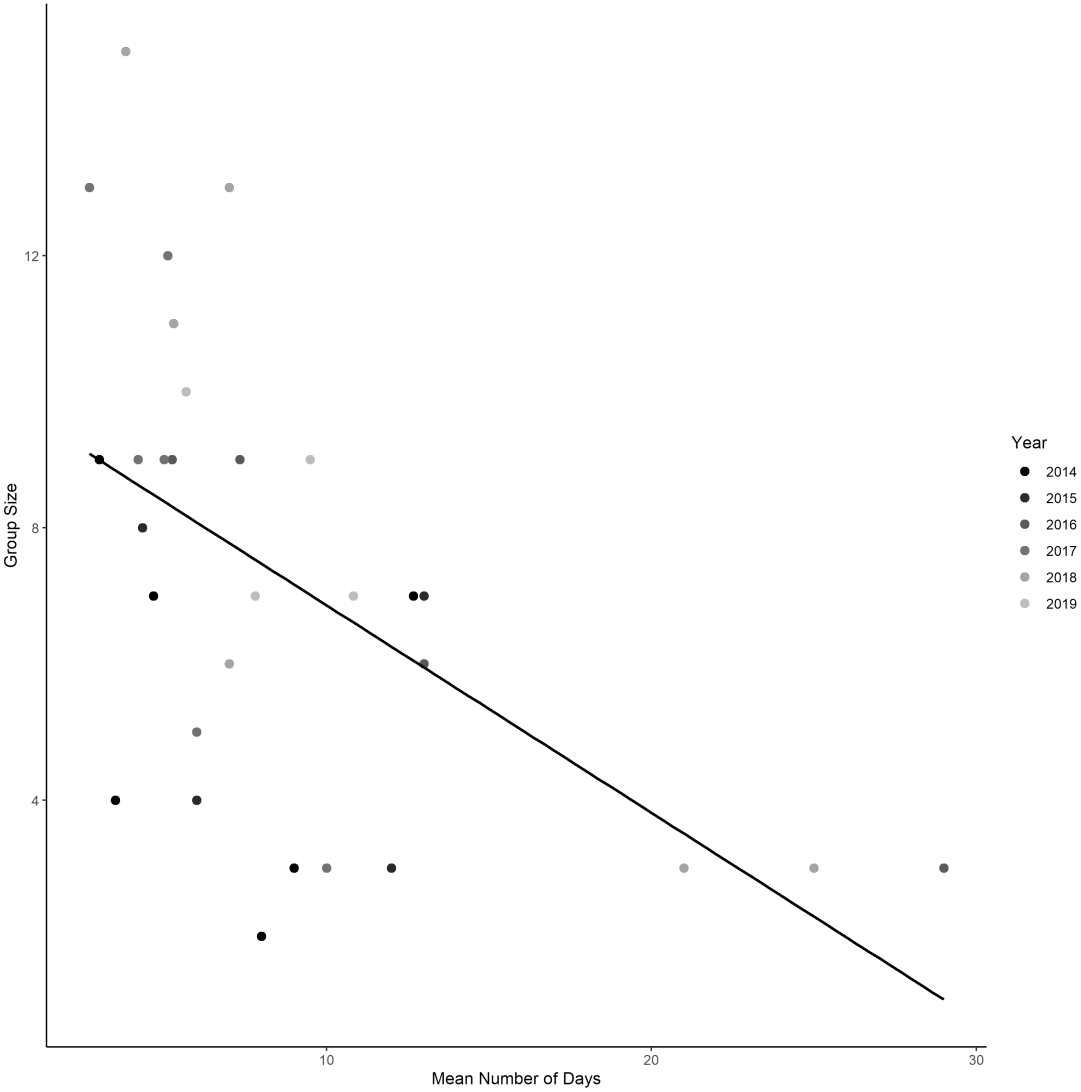
Dot plot of the mean number of days between first nest attempts of female eastern wild turkeys (*Meleagris gallopavo silvestris*) within groups relative to the size of each group on Kisatchie National Forest and Peason Ridge Wildlife Management Area, west central Louisiana, USA during 2014–2019. Plot suggests that larger social groups have more synchronous initial nest attempts than smaller groups.

Across all years, mean date of first nest initiation was 12 April (SE = 1.2, range = 12 March to 23 May, median = 10 April). The earliest mean date of first nest initiation for a group was 24 March (SE = 10.5, range = 14 March to 4 April, median = 24 March), whereas latest mean date was 6 May (SE = 4, range = 2–10 May, median = 6 May; Figure 2). We found that number of days between subsequent nest attempts was negatively (β = −0.993, SE = 0.0.285, *p* < .01) influenced by group size (Figure 3). For all years, there were 21 successful first nest attempts (14% nest success), of which 6 (29%) were the first nests initiated within a group, and the mean date of initiation for successful first nest attempts was 7 April. Approximately 30% of successful initial first nests were produced by 4% of females. We did not detect a statistical difference in nest success relative to date of nest initiation (β = −0.011, SE = 0.021, *p* = .58).

**FIGURE 4:**
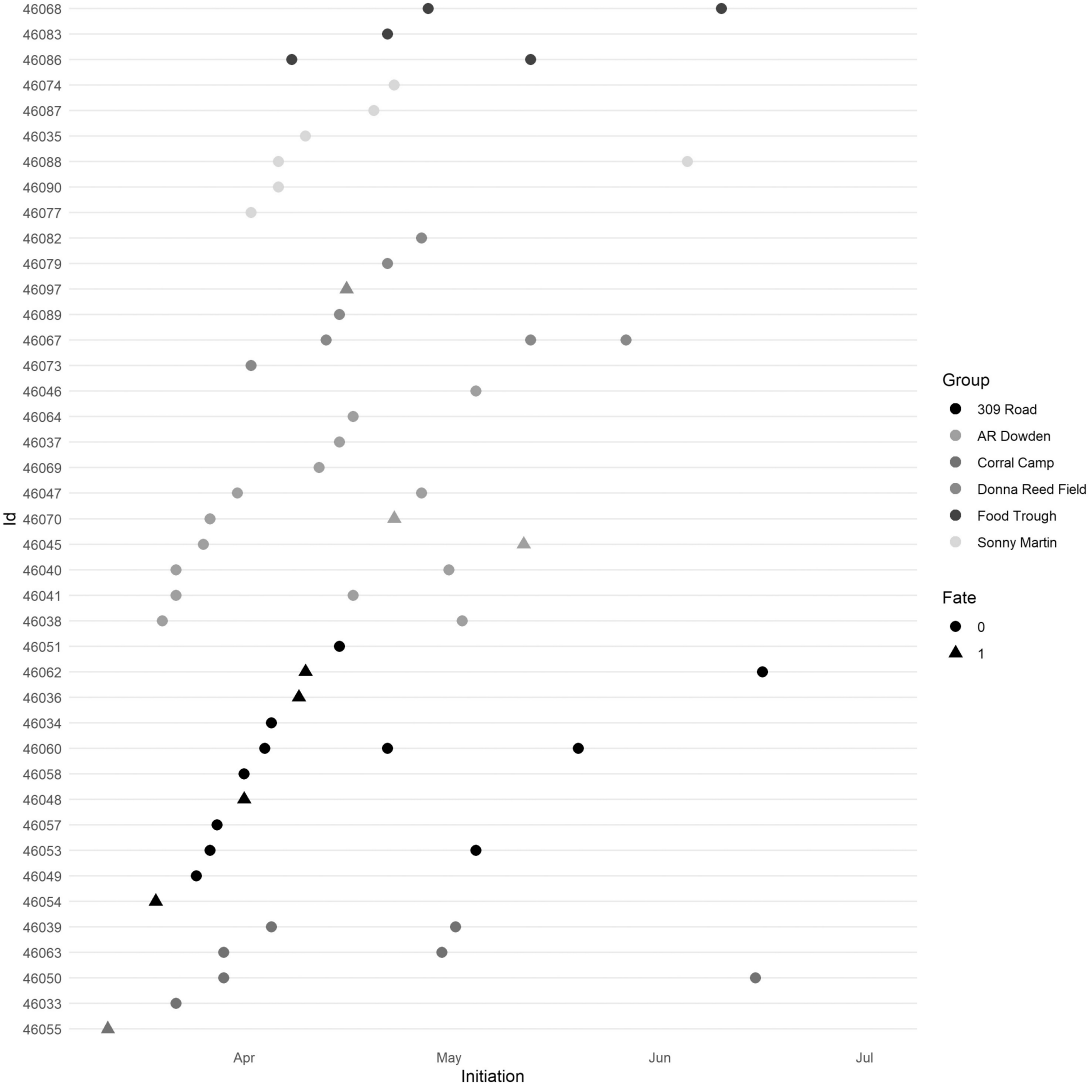
Dot plot of initiation dates for each individual female eastern wild turkey (*Meleagris gallopavo silvestris*) nest attempt within groups on Kisatchie National Forest and Peason Ridge Wildlife Management Area, west central Louisiana, in 2017.

We observed that the mean number of days between successive first nest attempts by females within a group varied from 3 to 7 days across years [mean = 3.9 (SE = 0.5) in 2014; mean = 3.0 (SE = 0.5) in 2015; mean = 7.4, (SE = 0.7) in 2016; mean = 4.2 (SE = 0.3) in 2017 (Supporting Information; Figure 4); mean = 4.6 (SE = 0.4) in 2018; means = 7.2 (SE = 0.5) in 2019]. Mean number of days between first nest attempts by females within a group was 49% less for successful (mean = 2.8, SE = 0.4) than failed (mean = 5.5, SE = 0.2) attempts (*z* = −4.51, *p* < .001).

## 4 DISCUSSION

Our current understanding of social behavior in wild turkeys is based on visual observations of interactions during the breeding season (Healy, 1992; Watts & Stokes, 1971). We used hourly GPS location data to assess social behaviors coupled with timing of nest initiation to provide an approach to evaluate reproductive asynchrony in wild turkeys (Bakner et al., 2019; Lohr et al., 2020). Our findings offer evidence that individual reproduction within female groups is asynchronous within wild turkeys and that reproductive success is influenced by timing of reproduction.

We found that prior to the onset of nest initiation, >80% of females within groups overlapped ranges. As individuals occupied shared space and were not territorial, our findings support the concept that female wild turkeys have stable flocks (Brown, 1975) maintained through social interactions (Healy, 1992; Watts & Stokes, 1971). Stable groups have been observed in multiple avian species, including female black grouse (*Lyrurus tetrix tetrix*) who frequently occupied the same territory while foraging (Kruijt & Hogan, 1967), groups of female greater prairie chickens that exhibited social interactions during visits to leks (Robel & Ballard Jr., 1974), and female sage grouse (*Centrocercus urophasianus*) maintained social hierarchies that influenced timing of reproduction (Scott, 1942).

The mean proportion of individual ranges prior to onset of initiation that did not overlap was 7.18%. We suspect that individuals that did not have overlapping ranges with groups we captured most likely belonged to a different flock that we did not capture. This suggests that these individuals may not have accessed the same males as those within the groups we monitored, and instead may have accessed different males, potentially skewing our results relative to asynchronous reproduction. However, only five of the 30 groups contained 1–2 individuals with ranges that did not overlap, which we contend would have produced minimal influences on our results.

We observed that timing of the onset of first nest initiation at the population level (e.g., across our study site) was similar across years. Similar observations have been previously noted and have attributed synchronous nesting behaviors at the population level to photoperiod influencing timing of reproduction (Healy, 1992; Migaud et al., 2006; Walton et al., 2011). The average dates of first nest initiation for female wild turkeys on our site were comparable to dates reported across populations throughout the southeastern United States, suggesting that the onset of nest initiation should occur during the 2‐week period we reported herein (Crawford et al., 2021; Keever et al., 2022; Palmer et al., 1993; Thogmartin & Johnson, 1999).

Within female groups, we found substantive temporal variation in timing of nest initiation within years. We observed that ~30% of successful initial first nests were produced by ~4% of females in our population. Previous works have noted the importance of successful early nesting attempts in sustaining wild turkey populations (Crawford et al., 2021; Porter et al., 1983). We did not find an effect of earlier nest initiation on nest success, but Keever et al. (2022) found strong evidence of reproductive benefits to earlier nesting attempts across the southeastern United States. Contemporary literature has detailed the ecological significance of females successfully hatching clutches early in the nesting season for various species, including willow ptarmigan (*Lagopus lagopus*; Wilson et al., 2007) and greater sandhill cranes (*Antigone canadensis*; Ivey & Dugger, 2008); however, these species tend to experience a shorter climatic seasonality compared to wild turkeys in the southeastern United States. We identified 15 instances of females whose initial nest failed, and they began laying their second nest attempt before remaining females‐initiated laying of their first nest attempt. However, we acknowledge that with our hourly GPS fixes there is a slight probability that we missed a first nest attempt that failed several days into the laying phase, and therefore misclassified a second nest attempt as a first nest attempt.

We expected 1–2 days to occur between each female within a group initiating their first nest attempt, based on observations of nesting behaviors in captive wild turkeys detailed by Healy (1992). However, we found that, on average, 3–7 days elapsed between successive nest attempts by individual females within a group. A nest was more likely to be successful if there was an average of 2.8 days between the initiation of another nest in their group. When two nests from a group were initiated with less than 3 days apart, one of the nests was more likely to be successful, compared to if two nests were initiated with greater than 5 days between them. We acknowledge that we had no way of confirming that we captured all individuals in each flock. Likewise, we also recognize that some individuals may parasitize nests instead of initiating their own nest, and therefore delay their nest attempt (Krakauer, 2008; Sullivan et al., 2022), which would potentially influence our results.

Robel and Ballard Jr (1974) noted that disruption of subordinate female greater prairie chickens by dominant females caused 2–3‐day delays in copulation and female–female aggressions are common in black grouse at leks (Karvonen et al., 2000). Disruption could increase time between successive nest attempts across females within a group, which may be important if there is a short time window for mating. Such disruption would ultimately benefit females that nest first if copulation was delayed in females waiting to copulate or if they were forced to mate with inferior males (Bro‐Jørgensen, 2002; Foster, 1981). However, female disruption only offers a partial explanation for the delay between successive nest attempts we observed. In lekking species, females are constrained in mate choice, and disruption of copulations between females and dominant males can occur via harassment from low‐ranking males, which also could delay nesting (Foster, 1983; Trail, 1985). Wild turkeys are primarily hunted during spring which overlaps with their breeding season and hunting coupled with male harvest is known to influence breeding behaviors of males (Chamberlain et al., 2018; Wakefield et al., 2020; Wightman et al., 2018, 2023). In polygynous species, only a few males are required to fertilize females, but removal of males may force females to travel farther to find suitable dominant males or return to breeding areas repeatedly to copulate with those same males, resulting in a delay in onset of nesting (Alatalo et al., 1988).

Our findings suggest that larger groups of females tended to exhibit more synchronized nest initiation. As group sized increased, the number of days between nest attempts across females declined, and more synchronized nesting attempts within a group (as determined by days between nest attempts across females) resulted in greater probability of nest success. Previous research has demonstrated that larger male coalitions of wild turkeys attract more breeding opportunities through improved mate attraction relative to smaller coalitions (Krakauer, 2008), which also has been observed in other species that use forms of leks (Jiguet & Bretagnolle, 2006; Ryder et al., 2009). Larger male coalitions contain the highest‐ranked males within the breeding population and are typically associated with larger groups of females (Bygott et al., 1979; Krakauer, 2005; Watts & Stokes, 1971). Conversely, smaller groups of females are often associated with pairs of subordinate males or singletons (Watts & Stokes, 1971). In species with pronounced social hierarchies, breeding with dominant males confers females with fitness benefits such as greater reproductive success (Majolo et al., 2012; Wong & Candolin, 2005), offering a partial explanation for our observation that nest success was greater in larger groups with decreased nesting asynchrony. Lastly, in birds where females aggregate into groups, reproductive success in general may be positively associated with group size (Burger, 1979; Canestrari et al., 2008).

Previous works noted that female wild turkeys typically nested within ~4 km of their pre‐laying range (Badyaev & Faust, 1996; Vander Haegen et al., 1988). Wild turkeys often shift ranges and exhibit temporal variation in resource selection as females move to breeding areas (Badyaev et al., 1996; Little et al., 2016; Miller & Conner, 2007). We found that distances between nest sites and the centroid of the 21‐day range before nest initiation failed to influence nest fate. Our finding contradicts Badyaev et al. (1996) who reported that females that traveled greater distances between pre‐nesting ranges and their eventual nest site had greater nest success.

Our findings suggest asynchronous reproduction is likely to occur in a male dominance polygynous mating system, which influences individual reproductive success. We found that individuals within groups shared space and stability within these flocks is maintained through social interactions (Healy, 1992). We propose that dominance hierarchies observed by Healy (1992) may influence the influence of timing of nest initiation in female wild turkeys. We suggest future research evaluate potential influences of sociality and dominance on nest success. As the genetic structure of social groups can have important implications for fitness and emerging evidence suggests that females identify a cost to associating with kin during the early reproductive season (Watkins, 2022), we suggest future research also include evaluating genetic variation across females within groups (Sugg et al., 1996).

## AUTHOR CONTRIBUTIONS

**Erin E. Ulrey:** Data curation (lead); formal analysis (supporting); investigation (supporting); methodology (supporting); software (supporting); visualization (supporting); writing – original draft (lead). **Michael J. Chamberlain:** Conceptualization (supporting); investigation (supporting); methodology (supporting); writing – original draft (supporting). **Bret A. Collier:** Conceptualization (supporting); funding acquisition (lead); methodology (supporting); project administration (lead); supervision (lead); writing – review and editing (supporting).

## CONFLICT OF INTEREST STATEMENT

None.

## Supporting information


Appendix S1.

## Data Availability

The data files used in all analyses along with metadata are available upon request or can be accessed on Dryad (10.5061/dryad.fbg79cp10).
