# Supplementary material for: Reproductive asynchrony within social groups of female eastern wild turkeys
Source: Ecol Evol. 2023 Jun 14;13(6):e10171. doi: 10.1002/ece3.10171 (PMC10266966; doi:10.1002/ece3.10171)
Supplement: Supplementary file 1 — Appendix S1. [file ECE3-13-e10171-s001.pdf]

Table A1. Number (No.) of female wild turkeys within each group (S), number of nest attempts (n), number of first (1), second (2), third (3), and fourth (4) nesting attempts, mean date of first nest initiation (MeanInit) with associated standard deviation in parentheses, median date of first nest initiation (MedInit), range of dates of first nest initiation (R), mean number of days between first nest attempts (Days) with associated standard deviation in parentheses, mean distance (Dist, m) between each nest location and the centroid of 99% utilization distributions 21 days prior to the first nest attempt on Kisatchie National Forest and Peason Ridge WMA in west-central Louisiana, USA during 2014–2019.

**Year**	**S**	**No.**	**n**	**1**	**2**	**3**	**4**	**MeanInit**	**MedInit**	**R**	**Days**	**Dist**
**2014**	1	9	9	5	4	0	0	4/23 (5.1)	4/21	4/17 – 4/29	3 (3.2)	2883
	2	7	7	4	2	1	0	4/13 (17.5)	4/10	3/28 - 5/5	12.7 (7.6)	3493
	3	7	6	4	2	0	0	4/23 (5.9)	4/23	4/17 – 5/1	4.7 (1.5)	983
	4	3	5	2	1	1	1	4/13 (6.5)	4/13	4/9 – 4/18	9	2895
	5	2	2	2	0	0	0	5/6 (5.7)	5/6	5/2 – 5/10	8	1234
	6	4	5	3	2	0	0	4/12 (3.5)	4/12	4/9 – 4/16	3.5 (0.7)	1767
**2015**	7	7	5	4	1	0	0	4/14 (17.2)	4/15	3/25 – 5/3	13 (3)	3754
	8	4	5	3	2	0	0	4/3 (6.4)	4/1	3/30 – 4/11	6 (5.7)	2709
	9	3	3	2	1	0	0	4/3 (8.5)	4/3	3/28 – 4/9	12	2093

	10	8	14	7	4	3	0	4/5 (8.7)	4/7	3/20 – 4/15	4.3 (3.3)	1912
**2016**	11	9	12	9	3	0	0	4/23 (16.2)	5/1	4/9 – 5/1	5.3 (6.0)	2228
	12	3	3	2	1	0	0	4/9 (20.5)	4/9	3/26 – 4/24	29	2681
	13	9	6	5	1	0	0	4/24 (10.4)	4/28	4/9 – 5/1	7.3 (8.1)	1697
	14	6	4	3	1	0	0	4/30 (14.5)	5/8	4/24 – 5/10	13 (15.6)	1293
**2017**	15	13	15	11	3	1	0	4/1 (7.9)	4/1	3/19 – 4/15	2.7 (2.0)	1179
	16	12	16	10	6	0	0	4/4 (15.1)	3/29	3/20 – 5/5	5.1 (6.0)	2741
	17	5	8	5	3	0	0	3/25 (9.1)	3/29	3/12 – 4/5	6.0 (4.2)	1940
	18	9	8	6	1	1	0	4/15 (8.5)	4/15	4/2 – 4/27	5.0 (3.9)	6403
	19	3	5	3	2	0	0	4/19 (10.3)	4/22	4/8 – 4/28	10.0 (5.7)	1421
	20	9	7	6	1	0	0	4/11 (8.5)	4/8	4/2 – 4/23	4.2 (3.6)	974
	21	6	8	5	3	0	0	4/10 (10.1)	4/8	3/29 – 4/26	7.0 (6.8)	1437
	22	13	12	7	4	1	0	4/19 (16.0)	4/26	3/27 – 5/8	7.0 (6.2)	1555
	23	11	10	8	1	1	0	4/22 (13.4)	4/22	4/8 – 5/15	5.3 (4.1)	1330
	24	3	3	3	0	0	0	3/24 (14.9)	3/24	3/14 – 4/4	21	1637

**2018**	25	15	19	12	5	2	0	4/14 (14.0)	4/12	3/29 – 5/10	3.8 (2.6)	1652
	26	3	3	2	1	0	0	4/22 (17.7)	4/22	4/10 – 5/5	25	3395
**2019**	27	10	14	7	4	3	0	4/6 (11.4)	4/6	3/21 – 4/24	5.7 (3.3)	2030
	28	7	10	7	2	1	0	4/5 (23.2)	3/26	3/19 – 5/23	10.9 (13.9)	2486
	29	7	14	6	6	2	0	3/30 (14.1)	3/27	3/16 – 4/24	7.8 (5.4)	2046
	30	9	7	5	2	0	0	4/10 (15.3)	4/3	3/28 – 5/5	9.5 (8.6)	1916

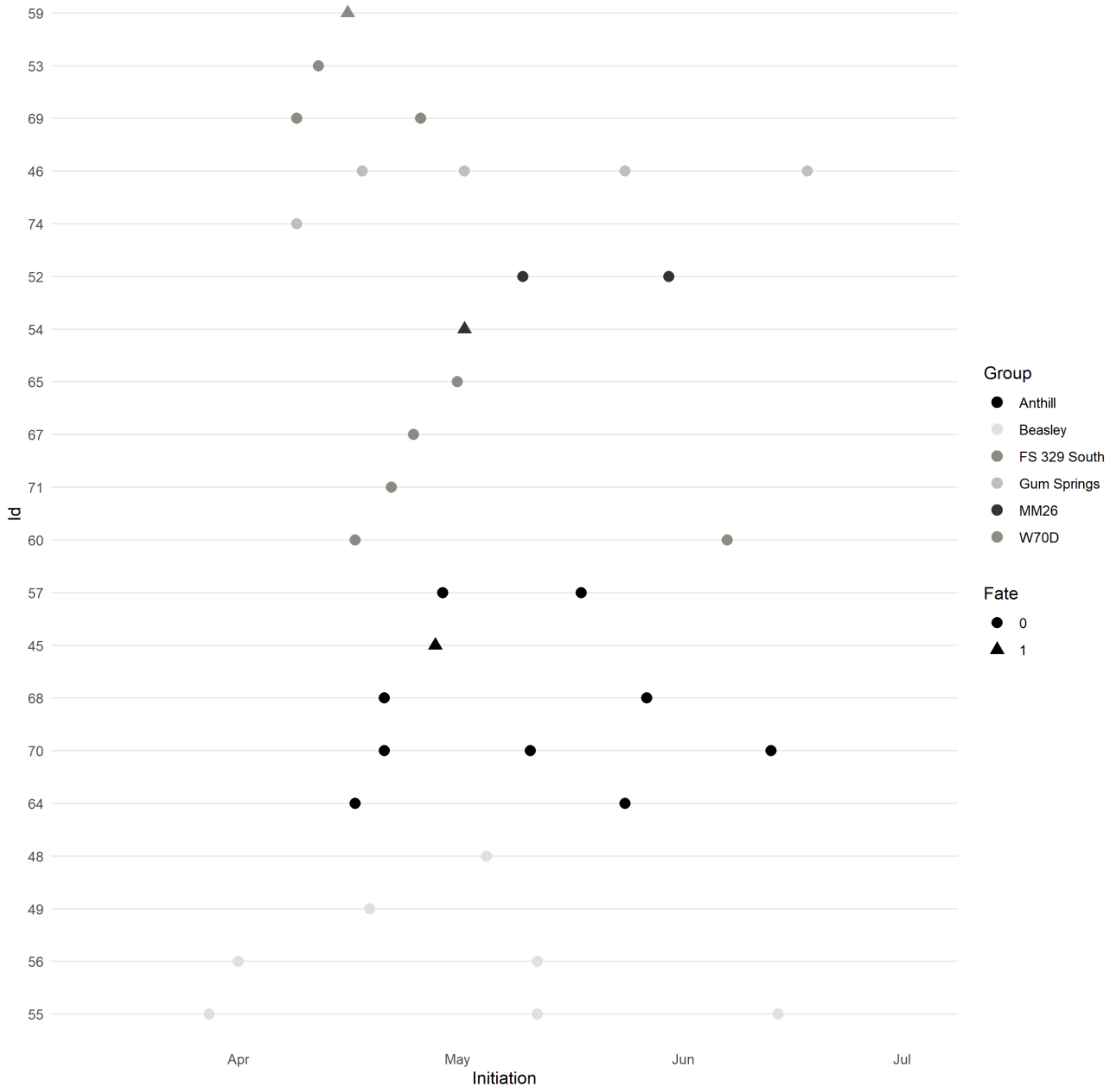

Figure A1. Dot plot of initiation dates for each individual female eastern wild turkey (*Meleagris gallopavo silvestris*) nest attempts within groups on Kisatchie National Forest and Peason Ridge Wildlife Management Area, west-central Louisiana, in 2014.

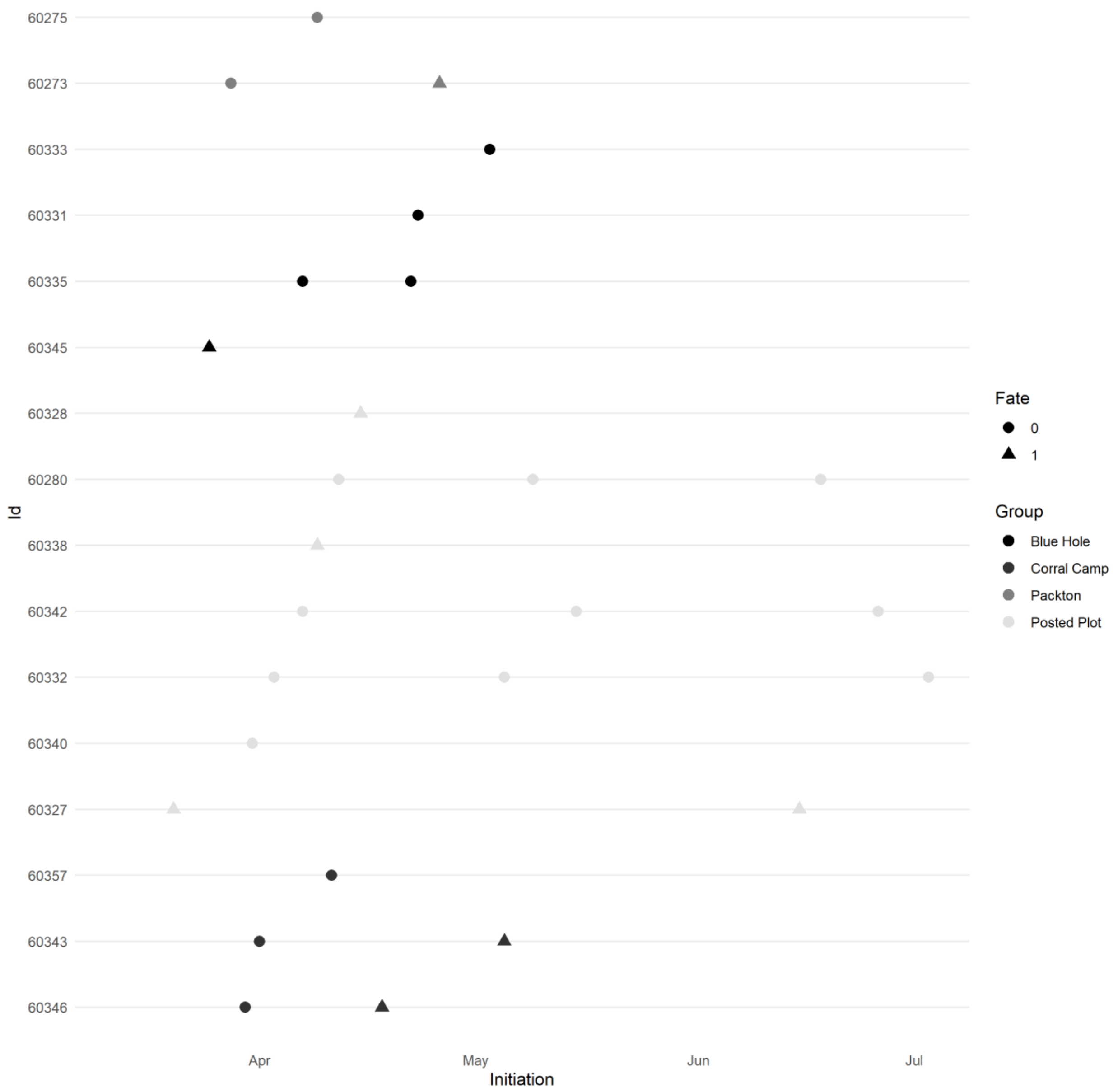

Figure A2. Dot plot of initiation dates for each individual female eastern wild turkey (*Meleagris gallopavo silvestris*) nest attempt within groups on Kisatchie National Forest and Peason Ridge Wildlife Management Area, west-central Louisiana, in 2015.

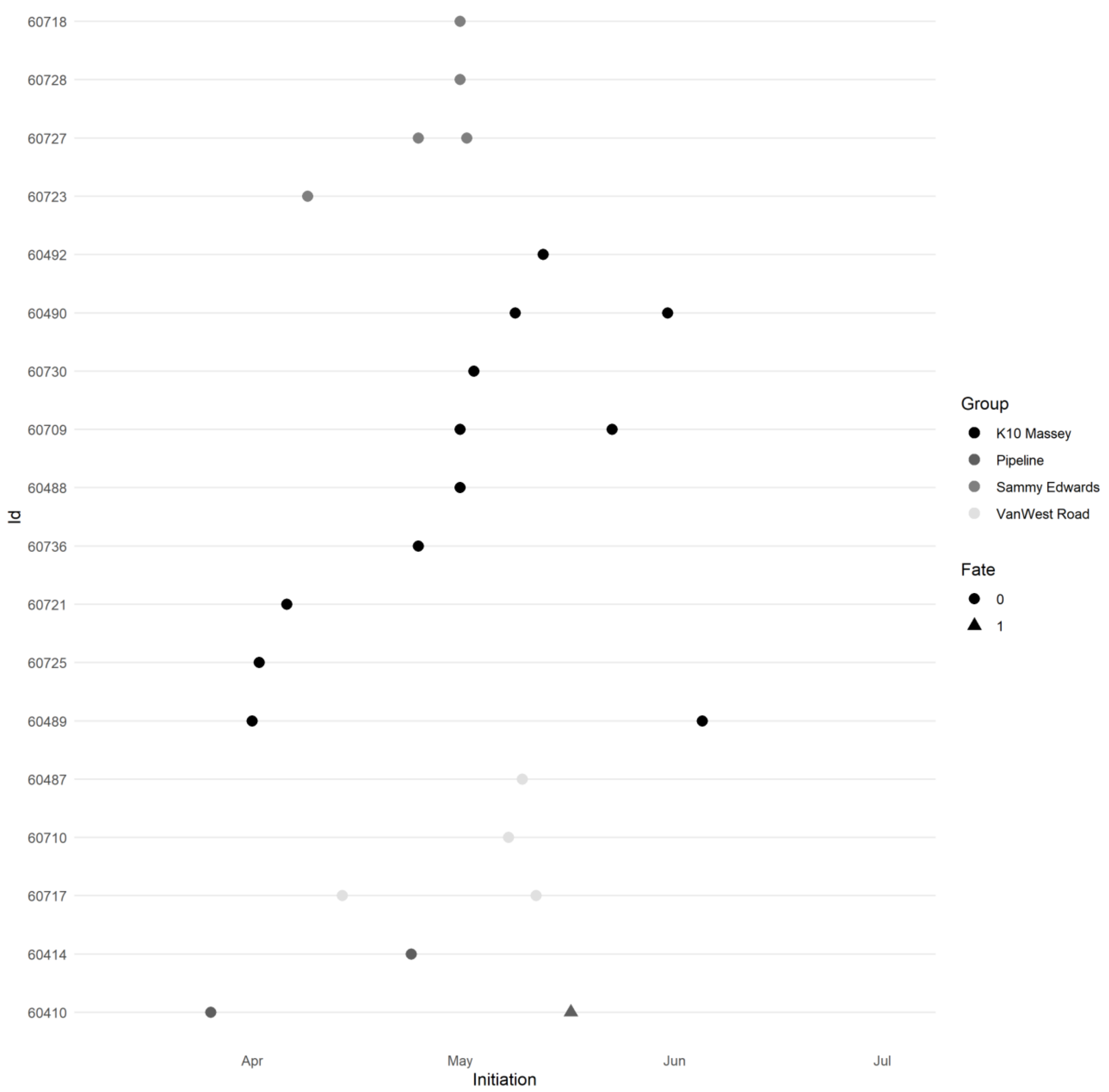

Figure A3. Dot plot of initiation dates for each individual female eastern wild turkey (*Meleagris gallopavo silvestris*) nest attempt within groups on Kisatchie National Forest and Peason Ridge Wildlife Management Area, west-central Louisiana in 2016.

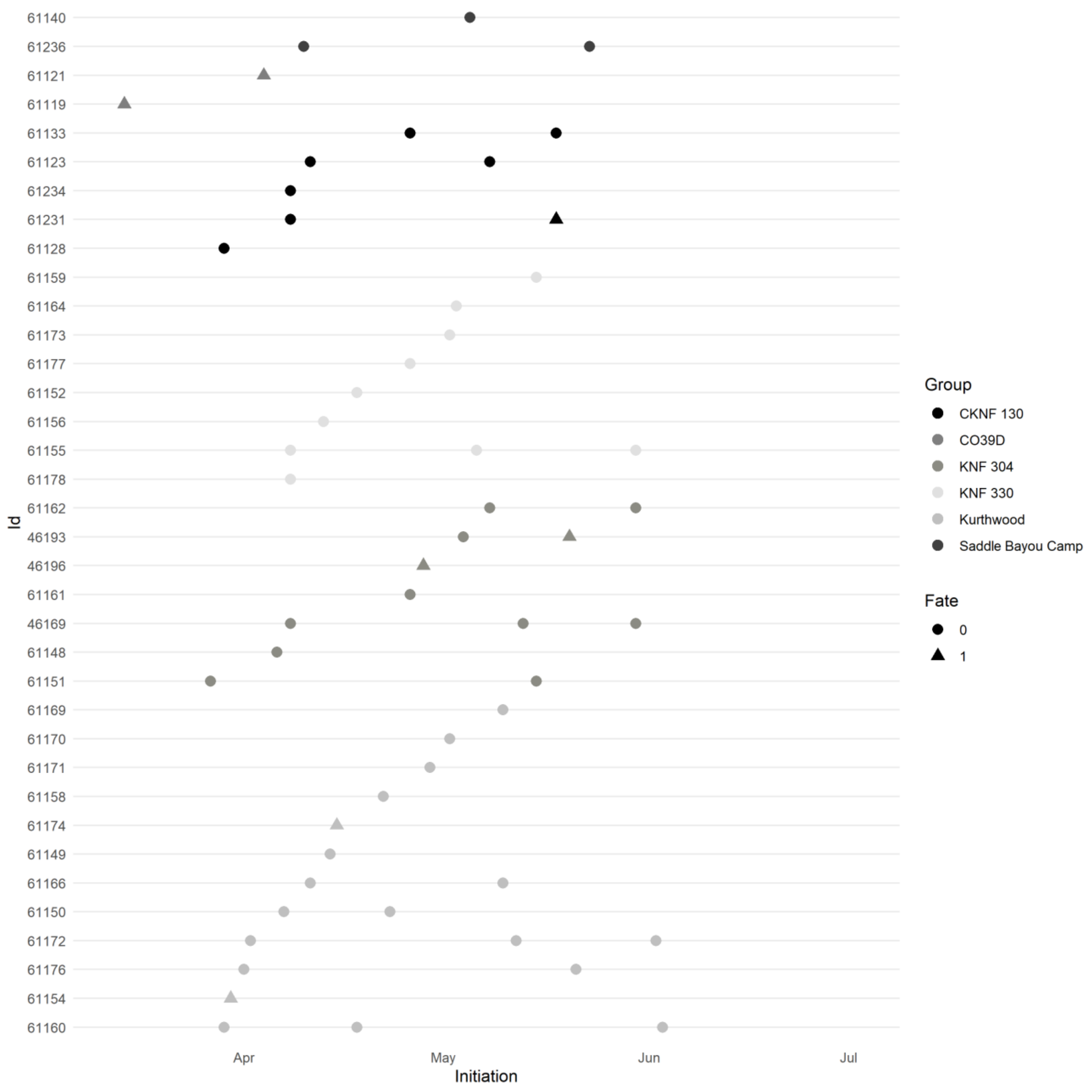

Figure A4. Dot plot initiation dates for each individual female eastern wild turkey (*Meleagris gallopavo silvestris*) nest attempt within groups on Kisatchie National Forest and Peason Ridge Wildlife Management Area, west-central Louisiana, in 2018.

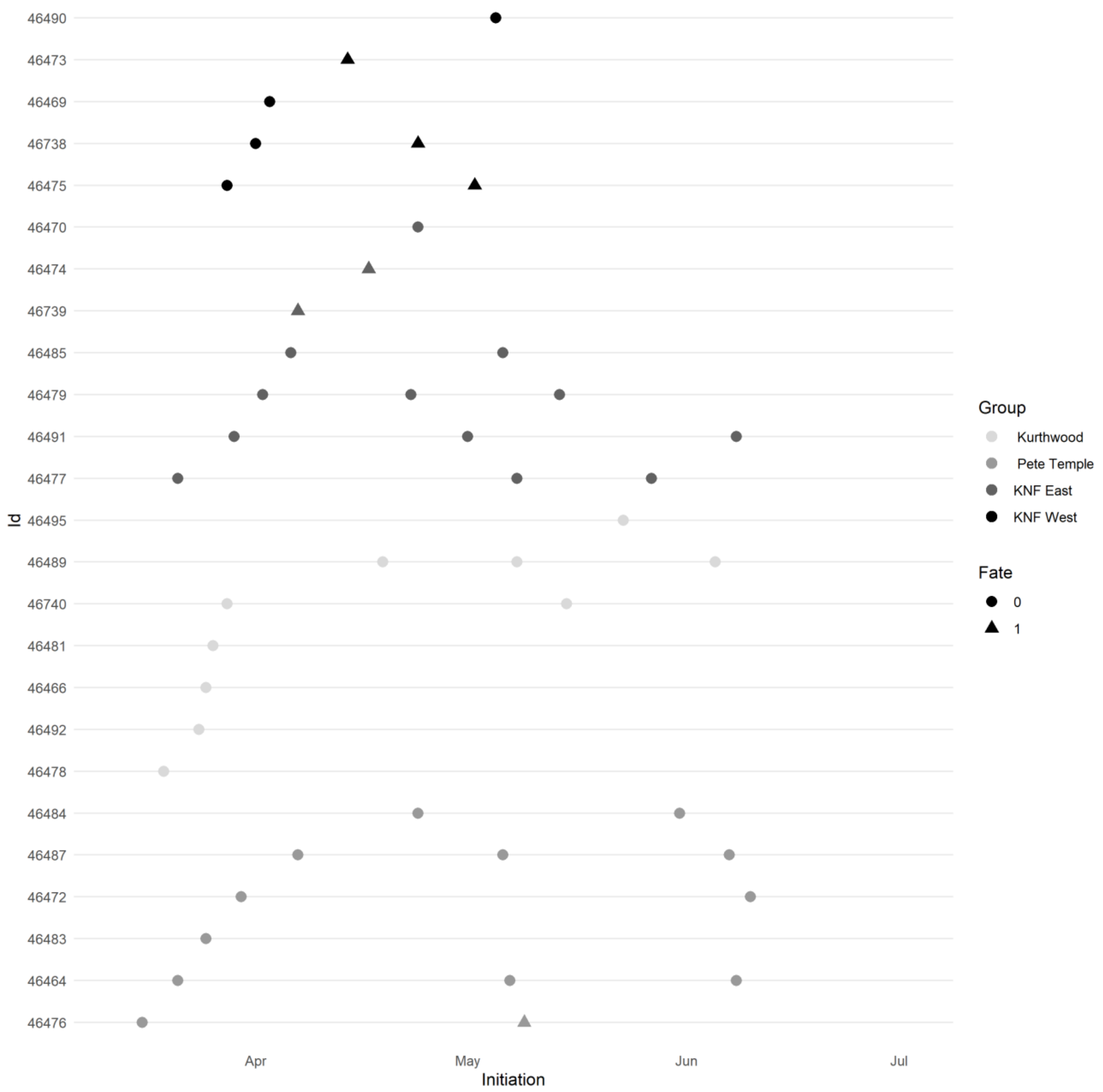

Figure A5. Dot plot of initiation dates for each individual female eastern wild turkey (*Meleagris gallopavo silvestris*) nest attempt within groups on Kisatchie National Forest and Peason Ridge Wildlife Management Area, west-central Louisiana, in 2019.
